# Socioeconomic inequality of cancer mortality in the United States: a spatial data mining approach

**DOI:** 10.1186/1476-072X-5-9

**Published:** 2006-02-15

**Authors:** Srinivas Vinnakota, Nina SN Lam

**Affiliations:** 1 Department of Geography and Anthropology, 227 Howe-Russell Geoscience Complex, Louisiana State University, Baton Rouge, LA 70803, USA

## Abstract

**Background:**

The objective of this study was to demonstrate the use of an association rule mining approach to discover associations between selected socioeconomic variables and the four most leading causes of cancer mortality in the United States. An association rule mining algorithm was applied to extract associations between the 1988–1992 cancer mortality rates for colorectal, lung, breast, and prostate cancers defined at the Health Service Area level and selected socioeconomic variables from the 1990 United States census. Geographic information system technology was used to integrate these data which were defined at different spatial resolutions, and to visualize and analyze the results from the association rule mining process.

**Results:**

Health Service Areas with high rates of low education, high unemployment, and low paying jobs were found to associate with higher rates of cancer mortality.

**Conclusion:**

Association rule mining with geographic information technology helps reveal the spatial patterns of socioeconomic inequality in cancer mortality in the United States and identify regions that need further attention.

## Background

From the application of a paper based point-map by John Snow to investigate the outbreak of cholera epidemic in 1854 to the current day practices of using digital maps, medical and public health profession has greatly benefited from the use of spatial information as manifested by maps. In this regard contemporary Geographic Information Systems (GIS) not only provide an excellent platform in which digital maps and data can be manipulated to extract valuable information, but also serve as an excellent medium for analyzing and visualizing spatial patterns. GIS and its associated spatial analysis have previously been used successfully to detect disease clusters [1,2], predict disease outbreaks [3,4], evaluate accessibility to health care facilities [5], determine health-environment interactions [6-8], and analyze spatial distribution of disease [9,10]. One such area has been the study of spatial distribution of cancer incidence and mortality [11].

Although successful applications of GIS and spatial analysis in cancer epidemiology have been made, limitations on the use of these methods have also been noted [12-14]. Recent spatial data mining methods that have been efficiently applied to mine large-scale data in other research fields could be useful to analyze cancer phenomena in a large scale [15-20]. The purpose of this study is to introduce the use of an association rule mining approach to uncover spatial associations between cancer mortality and socioeconomic characteristics in the United States, and identify regions that require further attention. Previous studies have shown how socioeconomic inequalities exist with respect to the distribution of cancer mortality using statistical techniques like principal components [21-23], regression [24,25], or by associating trends in mortality using data obtained from cancer registries [26,27] and surveys [28] with corresponding trends in socioeconomic characteristics. Statistical techniques like regression analysis provide for a global measure and require the dataset to be clean, non-noisy, and low dimensional, whereas association rule mining technique searches for locally occurring patterns in a very large, noisy and high-dimensional datasets [29-31]. While most exploratory spatial data analysis and visualization techniques can identify patterns or trends in data, they often perform poorly when the number of variables in the dataset is large [32]. For example, if a dataset has n attributes each consisting of m categories then there can exist  q possible combinations, involving k (k ≤ n) attributes in a combination, for association rules to be formed. Association rule mining is an efficient technique that sifts through all the possible combinations to extract frequently occurring patterns that satisfy user specified criteria. Association rules can be construed as *filters *that can be used to generate hypothesis for a more rigorous statistical analysis.

We focused on cancer mortality in this study because, among other reasons, its disparity among different populations is a serious concern that affects our social and economic well-being. Cancer is a family of diseases and there is no one cause or cure for cancer [33]. In the year 2002 cancer had been the second leading cause of death in the United States of America [34]. Some kinds of cancer have long been suspected of being genetically linked [35], while others are more prevalent in certain racial and ethnic groups than others [12]. It was argued that that the differences in cancer incidence and mortality rates that exist among racial and ethnic groups are probably the result of socioeconomic status rather than genetic and cultural aspects of race and ethnicity [26,27,36]. For example, associations between occupational exposure and prostate cancer were found [37-39], but with a higher incidence of cancer among African-American workers reported, reflecting racial disparities in levels of exposure to occupational carcinogens [40]. Breast cancer mortality rates among U.S. women decreased in areas of higher socioeconomic status while during the same time-period it increased in areas of lower socioeconomic status [41].

From a public health point of view it then becomes imperative that one has to assess the differences in socioeconomic, environment, and disease characteristics to find explanations. This study analyzes at a national level if socioeconomic inequality exists for the four most common cancers in the United States, including colorectal, lung, breast, and prostate cancers, using mortality rates aggregated at the Health Service Areas (HSA) level. With few exceptions [21-23,42,43], reports on the spatial distribution of socioeconomic inequality in cancer mortality at a national level have seldom been made. Although the unit of analysis in this study is not at a finer spatial resolution (e.g., county level), this study should provide useful information on where and how health disparity distributes at a national level, and whether association rule mining is a useful approach to derive such information.

## Results

**Figure 1:**
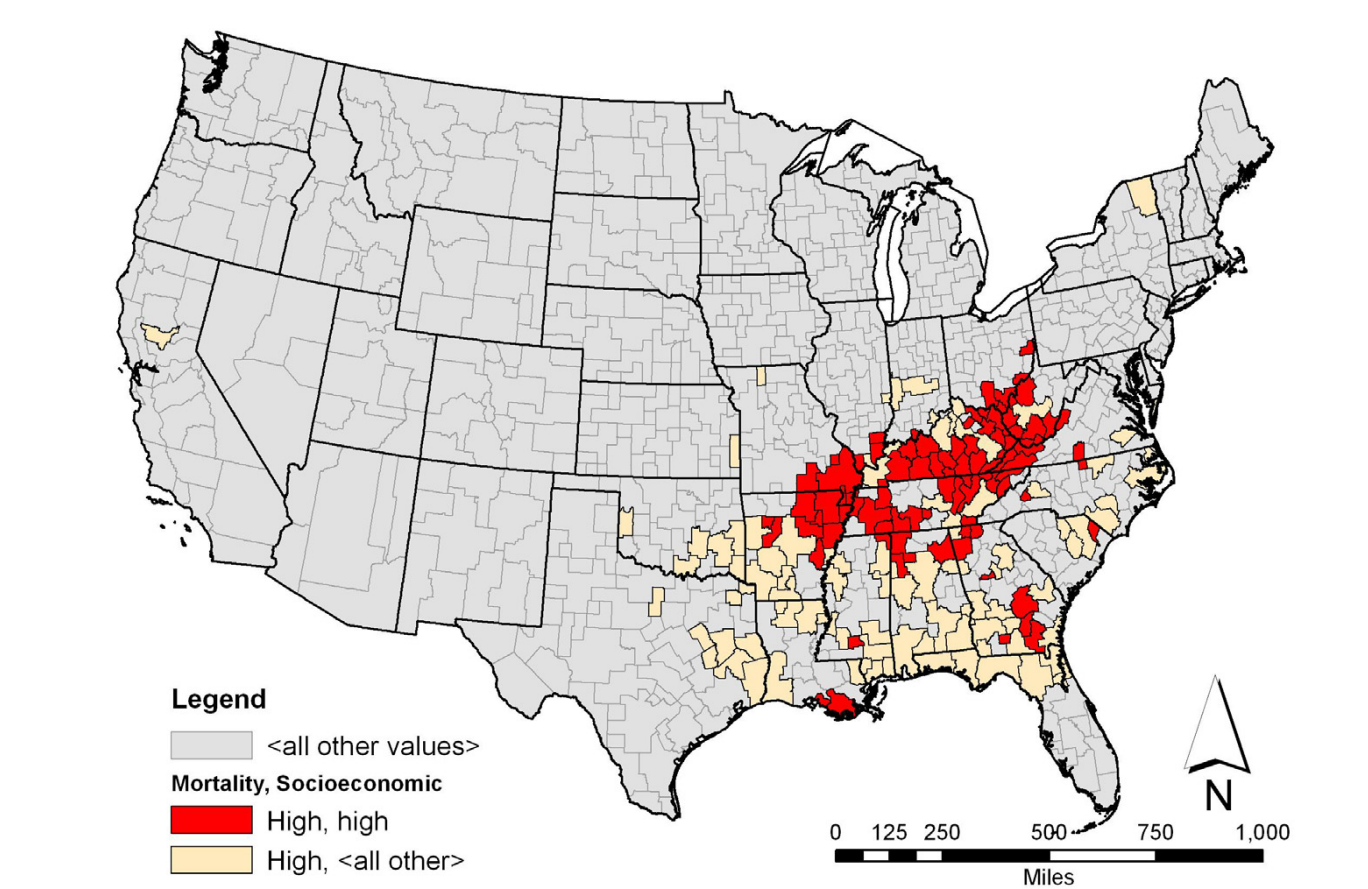
**Lung cancer mortality among white men and associated socioeconomic characteristic with the highest support**. Shown in red is the spatial distribution of areas having a high rate of lung cancer mortality among white men and high density of whites with low educational attainment. This pattern has the highest support among the association rules involving lung cancer mortality rate. The beige color indicates areas having high rate of lung cancer mortality among white men.

**Figure 2:**
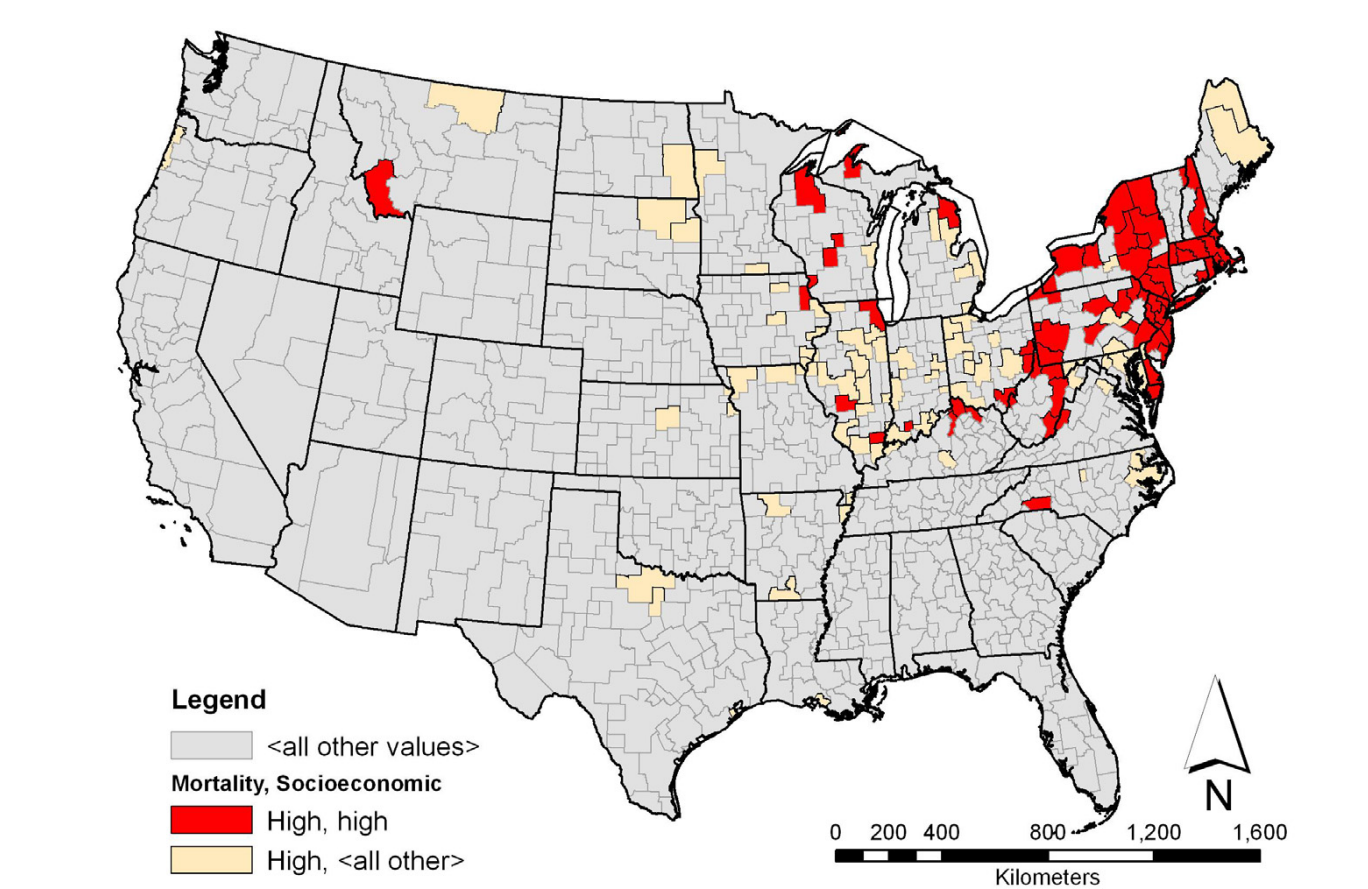
**Colorectal cancer mortality among white men and associated socioeconomic characteristic with the highest support**. Shown in red is the spatial distribution of areas having a high rate of colorectal cancer mortality among white men and high density of households with white male householder and no wife present and no children under the age of 18 years. This pattern has the highest support among the association rules involving colorectal cancer mortality rate. The beige color indicates areas having high rate of colorectal cancer mortality among white men.

**Figure 3:**
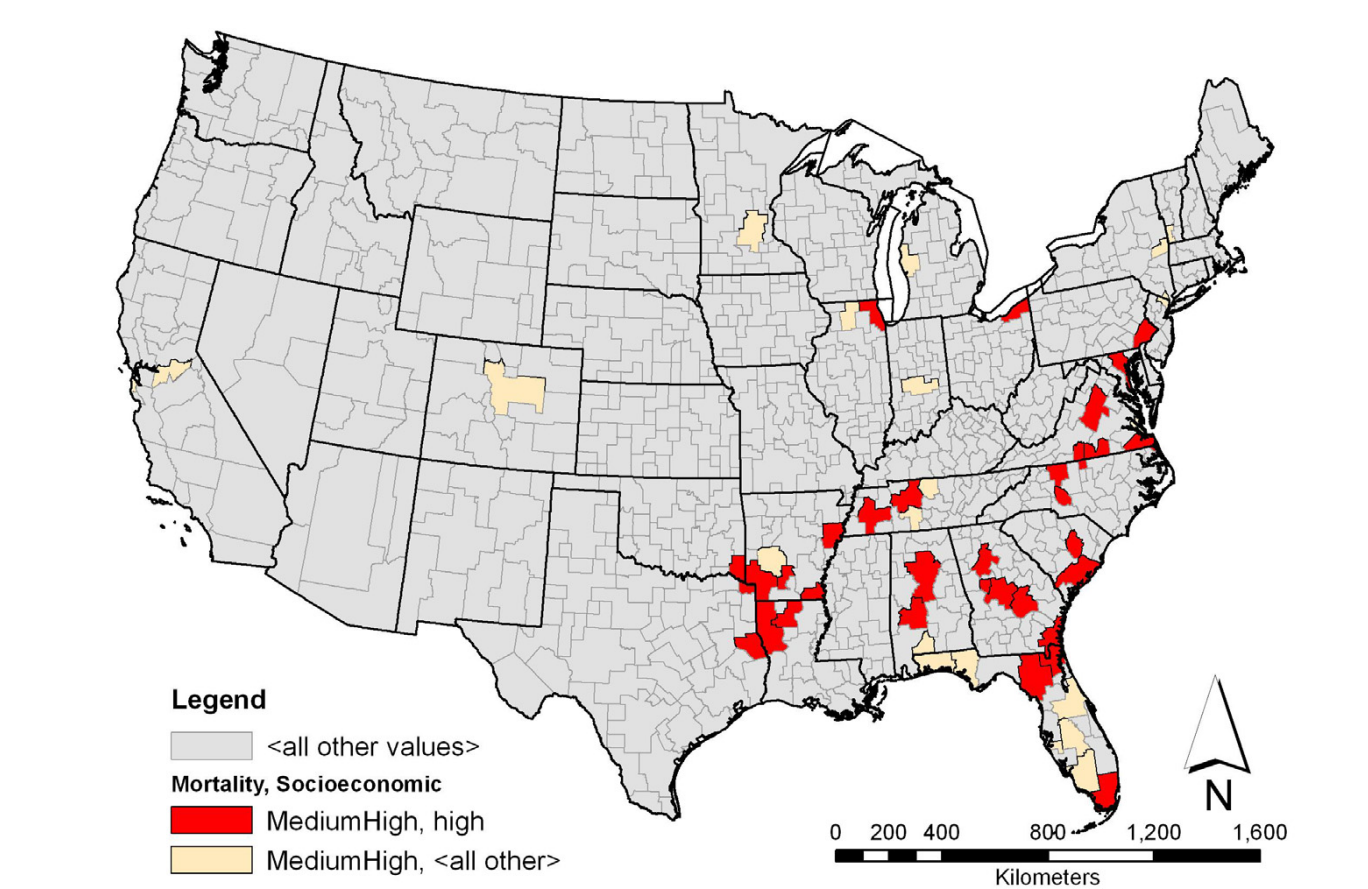
**Prostate cancer mortality among black men and associated socioeconomic characteristic with the highest support**. Shown in red is the spatial distribution of areas having a medium-high rate of prostate cancer mortality among black men and high density of households with black female householder with no husband present and with no children under the age of 18 years. This pattern has the highest support among the association rules involving prostate cancer mortality rate. The beige color indicates areas having medium-high rate of prostate cancer mortality among black men.

**Figure 4:**
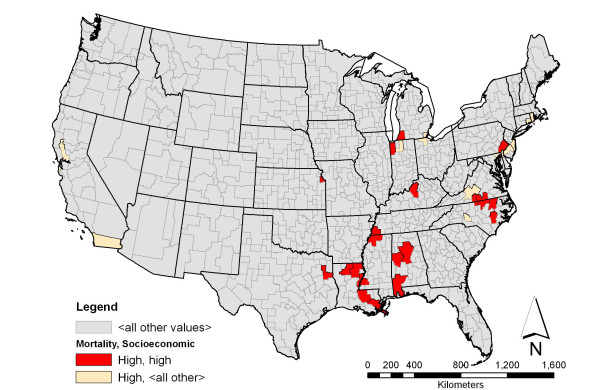
**Breast cancer among black women and associated socioeconomic characteristic with the highest support**. Shown in red is the spatial distribution of areas having high rate of breast cancer mortality among black women and high density of households with black female householder with no husband present and with the presence of own children under the age of 18 years. This pattern has the highest support among the association rules involving breast cancer mortality rate. The beige color indicates areas having high rate of breast cancer mortality among black women.

A total of 3002 association rules were generated using the specified minimum support (3%) and confidence (40%) values, of which only 426 involved cancer mortality rates in the antecedent part of the rule. Since the intent of this research was to study the socioeconomic characteristics of areas based on cancer mortality, rules that had associations among the socioeconomic variables themselves or rules that had the cancer mortality rate occurring in the consequent part of the rule were ignored. For each cancer type, we selected the rule that has the highest support value and reported in Table 5 (rules *a -d*), with the top three HSAs in each rule listed in Table 6. Figures 1, 2, 3, 4 map the HSAs identified in each of the four rules along the cancer mortality rate identified in the rule.

Association rule *a *identifies that of the 155 areas that had high rate of lung cancer mortality among white men, 72 exhibited high rate of low educational attainment among the white population aged 18 and above. HSAs generated from this rule were mostly concentrated in the Appalachian region (Figure 1). The top three HSAs in this rule based on lung cancer mortality among white male were: Madison, MO, Scott, TN, and Pike, KY – Logon, WV (Table 6).

Rule *b *associates areas with high rate of colorectal cancer mortality among white men with high density of family households having a white male householder and with no wife and no children under the age of 18 present in the same household. Sixty of 131 HSAs that had high rate of colorectal cancer mortality exhibit the pattern shown in rule *b*. Figure 2 maps the spatial distribution of the association rule pattern. HSAs that fit this rule were mostly located in the mid-west and northeast, with the top three HSAs being Jefferson, OH – Harrison, OH, White, IL – Hamilton, IL, and Lackawanna (Scranton, PA) – Wayne, PA (Table 6).

Association rule *c *describes areas with medium-high rate of prostate cancer mortality among black men as the areas that also had high density of households with a black female householder with no husband and no children under the age of 18 in the same household. At a confidence level of 61.22%, of the 49 HSAs that had medium-high rate of prostate cancer mortality among black men, thirty were associated with the socioeconomic variable presented in rule *c*. Figure 3 maps the spatial distribution of the association rule pattern. Most of the HSAs from this rule were found scattered in the south and southeast, with the top three being Dallas, AL – Marengo, AL, Ouachita, AR – Dallas, AR, and Upson, GA – Lamar, GA.

Association rule *d *describes health service areas that had high rate of breast cancer mortality among black females as the areas that also exhibited high density of households with a black female householder with no husband present and with the presence of children under the age of 18 in the household. Although rule *d *does not meet the minimum support value (of 3%) specified initially, it was chosen because it had the highest support among all the rules that contained breast cancer mortality in the antecedent. Despite its low support value, 23 HSAs out of the 31 areas that had high breast cancer mortality among black women were associated with the socioeconomic variable shown in the rule, yielding a high confidence value of 74.19%. HSAs that fit this rule were mostly in the south and parts of the east, with the top three being Halifax, VA – Mecklenburg, VA, Lincoln, LA – Union, LA, and Gregg (Longview), TX – Rusk, TX. Figure 4 maps the spatial distribution of the association rule pattern.

We only report 4 of the 426 rules extracted based on the specified minimum support and confidence values. In general, a reading of the rules that have high support values indicate that areas with high rates of cancer mortality were associated with low educational attainment, high rates of unemployment, higher percentage of the population employed in construction, mining, transportation, and agricultural industries, high density of households with no plumbing and no vehicles, and low percentage of the population employed in services and financial industries.

A correlation analysis performed on the cancer mortality rates and socioeconomic variables using Pearson's product-moment correlation produced 13 correlations that were moderate (r > plus or minus 0.40) and statistically significant (p < 0.01). Table 4 lists these correlations among the variables along with their corresponding correlation coefficient. The highest positive correlation coefficient occurred between colorectal cancer mortality among black women and unemployment rate among black female (r = 0.514), and the highest negative correlation was between lung cancer mortality among black women and percentage of widowed women living in an area (r = -0.468).

**Table 1:** Data variables used in association rule mining of cancer mortality rates and socioeconomic characteristics

Dataset	Variable	Source
Cancer mortality rates	Lung cancer among white/black men/women. Colorectal cancer among white/black men/women. Breast cancer among white/black women. Prostate cancer among white/black men.	All cause mortality shapefile from National Atlas of United States of America.
Socioeconomic characteristics	Number of persons in household: Low household density: Number of persons in a household is less than or equal to 3. High household density: Number of persons in a household is more than 3. Race of householder by household type and presence and age of children. Sex by marital status. Place of birth. Place of work. Race by educational attainment: Low education: Less than or equal to high school. High education: Any degree obtained beyond high school level. Race by sex by employment status. Poverty status in 1989 by race. Race of householder by vehicles available. Plumbing facilities. Unemployment rates. Number of persons employed in mining, service, occupation, manufacturing, retail, wholesale, construction, ag-services, and transportation industries.	United States Census Bureau: 1990 Census United States Bureau of Economic Analysis: 1988–1992

**Table 2:** Descriptive statistics for cancer mortality rates used in the analysis. Mortality rates reported are per 100,000 age-, sex-, and race-adjusted to 1940 standard US population

Cancer mortality rate	Number of records	Minimum	Maximum	Mean	Standard Deviation
White male lung cancer	774	9.41	119.29	58.9193	14.46512
White male colorectal cancer	654	7.52	25.46	16.1918	3.07422
White male prostate cancer	730	6.46	27.2	15.218	2.53059
White female lung cancer	684	8.61	49.36	24.7711	5.65126
White female breast cancer	699	9.46	37.31	21.5052	3.23601
White female colorectal cancer	622	4.3	21.18	11.0973	2.12812
Black male lung cancer	290	0	245.2	88.2525	20.70256
Black male colorectal cancer	114	0	36.89	21.3869	4.7887
Black male prostate cancer	244	0	61.38	35.7475	8.09248
Black female lung cancer	153	0	56.06	24.8152	9.62457
Black female breast cancer	158	0	43.21	26.3151	7.58142
Black female colorectal cancer	131	0	29.48	15.0637	4.82983

**Table 3:** Descriptive statistics for selected socioeconomic variables used in the study. Except for unemployment rates that expressed as a percentage the rest of variables are proportion of their corresponding universe

Socioeconomic variable	Number of HSAs	Minimum	Maximum	Mean	Std. Deviation
White male householder with no wife present and with no own children under the age of 18 years.	804	0	0.03	0.00969	0.001735
Black female householder with no husband present and with presence of own children under the age of 18 years in the same household	804	0	0.13	0.01279	0.020477
Black female householder with no husband present and with no own children under the age of 18 years	804	0	0.08	0.00757	0.012746
Women who are never married	804	0.08	0.77	0.1991	0.060248
Women who are married and now living with their spouse	804	0.31	0.86	0.5731	0.064284
Women who are widowed	804	0.05	0.37	0.13571	0.03033
Women who are divorced	804	0.008	0.3	0.08327	0.025265
Population born in the south but now living elsewhere	804	0.004	0.38	0.08357	0.062408
Population working in the same county as residence	804	0.24	0.7	0.44745	0.077879
Households with complete plumbing facilities	804	0.64	0.99	0.97546	0.021435
Households with no plumbing facilities	804	0.001	0.35	0.01678	0.020128
Black female unemployment rate	804	0	100	11.93057	11.867286
Population employed in services industry	804	0	0.88	0.27529	0.078398
Population employed in agricultural – services industry	804	0	0.23	0.01511	0.01745
White with less education attainment	804	0.23	0.85	0.59597	0.097935
White with high educational attainment	804	0.14	0.76	0.39404	0.097948

## Discussion

This study has demonstrated the use of association rule mining in revealing major spatial patterns of socioeconomic inequality of cancer mortality in the United States. We also call attention to some of the problems and future methodological improvements regarding the use of this method for public health applications. The results presented in the previous section are area-based measures of cancer mortality and socioeconomic characteristics. Ecological results such as these should be interpreted accordingly to avoid ecological fallacy. For example, the first association rule listed in Table 5a does not imply high occurrence of lung cancer among white men is necessarily caused or associated with individual's low educational attainment. It only indicates that areas that tend to have high rates of lung cancer mortality among white men also exhibit high rates of low educational attainment among whites. Whether cancer mortality and socioeconomic characteristics are causally related will need to be investigated separately. The association rules shown in Table 5 can be construed as a hypothesis for further research at a much finer granularity to investigate the correlation among the predicates of the rule. Incorporating knowledge from external sources in examining the rules is needed to fully interpret the results.

Knowledge discovery using association analysis is an iterative procedure at times requiring expert domain knowledge. Since the intent of the analysis is to extract implicit patterns from among the variables in the dataset, different combinations of support and confidence values have to be tested. Association rules might sometime exhibit strong measures of support, confidence, and lift values but still not provide any useful information. Some basic domain knowledge regarding the dataset might help overcome this limitation. The main drawback of association analysis is that it does not provide a statistical measure for the rules. On the other hand a similar argument in favor of association analysis is that it makes no assumption of the data being independent and identically distributed as is required by most statistical analysis. Since the process of association rule mining is data driven in which a pattern is induced based on the available data while making no assumption about the extracted pattern, this makes it an exploratory approach. The patterns extracted using association rule mining could be used to generate a hypothesis that could then be tested using statistical techniques.

A selected subset of rules based on correlation analysis and association rule mining involving white male lung cancer mortality rates are shown in Table 7. The support and confidence values for a rule is dependent on the number of data records included in the analysis; in case of public health datasets difference in the values can be observed based on whether all data records are included or only those records that report reliable rates are included. The lift value in an association rule signifies the probability of the consequent (socioeconomic variable) occurring given an occurrence of the antecedent (mortality rate). The low positive lift values in this case indicate that the consequent is likely to occur given an occurrence of the antecedent. While the statistical correlation analysis produced correlations among black female mortality rates and socioeconomic variables that were moderate (r > 0.4) and statistically significant, the association analysis did not discover any rules involving black female mortality rates at the specified support (3%) and confidence values (40%). This could either be due to the fact that discretization creates categories that are summaries of the continuous data representing only coarse information while leaving behind critical details in the dataset or such association rules might occur at less than the specified support and confidence values.

**Table 4:** Results of correlation analysis between cancer mortality rates and socioeconomic variables using Pearson's product-moment correlation

Cancer mortality rate	Socioeconomic variable	Correlation coefficient
Black female lung cancer	Women who are widowed	-0.468
Black female lung cancer	Women who are divorced	0.449
Black female lung cancer	Houses that have complete plumbing	0.438
Black female breast cancer	Women who are never married	0.438
Black female breast cancer	Women who are married and living with their spouse	-0.418
Black female breast cancer	Black female unemployment rate	0.486
Black female colorectal cancer	Women who are never married	0.422
Black female colorectal cancer	Black female unemployment rate	0.514
Black female colorectal cancer	Population employed in Agricultural services industry	-0.435
White male lung cancer	Population born in the south but living elsewhere	0.451
White male lung cancer	Population working in the same county as residence	-0.455
White male lung cancer	White population with less educational attainment	0.458
White male lung cancer	White population with high educational attainment	-0.458

**Table 5:** Association rules that have the highest support value for each of the four cancer sites used in the study

	Association Rule	Support%, Confidence%, lift
a	Lung cancer mortality among white men is high → proportion of whites with low educational attainment is high	8.955%, 46.45%, 2.32 (72/155)
b	Colorectal cancer mortality among white men is high → proportion of households with white male householder with no wife present and with no children under the age of 18 years is high	7.463%, 45.80%, 2.29 (60/131)
c	Prostate cancer among black men is medium high → proportion of households with black female householder with no husband present and with no children under the age of 18 years is high	3.731%, 61.22%, 3.06 (30/49)
d	Breast cancer among black females is high → proportion of households with black female householder with no husband present and with the presence of own children under the age of 18 years in the house is high	2.861%, 74.19%, 3.59 (23/31)

**Table 6:** Top 3 HSAs ordered on the cancer mortality rate for the association rules based on the highest support value

Rule	HSA	Mortality rate	Socio-economic variable value
a: Lung cancer mortality among white men is high → Whites with low educational attainment is high	Madison, MO	119.293	0.787
	Scott, TN	115.034	0.839
	Pike, KY – Logan, WV	100.694	0.801
b: Colorectal cancer mortality among white men is high → density of households with white male householder with no wife present and with no children under the age of 18 years is high	Jefferson (Steubenville), OH – Harrison, OH	24.503	0.017
	White, IL – Hamilton, IL	24.427	0.014
	Lackawanna (Scranton), PA – Wayne, PA	24.157	0.021
c: Prostate cancer among black men is medium high → density of households with black female householder with no husband present and with no children under the age of 18 years is high	Dallas, AL – Marengo, AL	41.445	0.074
	Ouachita, AR – Dallas, AR	41.364	0.033
	Upson, GA – Lamar, GA	41.017	0.035
d: Breast cancer among black females is high → density of households with black female householder with no husband present and with the presence of own children under the age of 18 years in the house is high	Halifax, VA – Mecklenburg, VA	39.206	0.041
	Lincoln, LA – Union, LA	38.517	0.051
	Gregg (Longview), TX – Rusk, TX	36.581	0.028

**Table 7:** Comparison between selected results of statistical correlation analysis and association rule mining

Type	Rule	Rule value: correlation coefficient for correlation rule and (support, confidence, lift) for association rule.
Correlation rule	White male lung cancer and percent population working in the same county as residence	-0.455
Correlation rule	White male lung cancer and percent white population with less educational attainment	0.458
Correlation rule	White male lung cancer and percent white population with high educational attainment	-0.458
Association rule	White male lung cancer mortality is low → percent population working in the same county as residence is high	8.706, 45.45%, 2.27
Association rule	White male lung cancer mortality is high → percent white with less educational attainment is high	8.955%, 46.45%, 2.32
Association rule	White male lung cancer mortality is high → percent white population with high educational attainment is low	8.831%, 45.81%, 2.30

One of the critical aspects of association rule mining is its requirement of categorical data. Since the categorical combinations of the attributes depend on the discretizing method and on the number of classes chosen to represent the data, it would be necessary for future studies to study the effects of these class interval selections on the results of association rule mining. Also, it is generally accepted that cancer mortality varies significantly with environmental conditions [44]. As such future studies should incorporate environmental data in addition to socioeconomic data. Finally, more detailed level data (e.g., counties, census tracts) are needed for more accurate analysis. Also, more variables can be included (e.g. median income) to better reveal the spatial distribution of cancer mortality and their associations with socioeconomic and environmental characteristics.

## Conclusion

This study demonstrates the application of association rule mining to extract patterns of spatial distribution of cancer mortality with respect to socioeconomic characteristics of health service areas. Since HSAs are at a very coarse scale we did not attempt to derive any hypothesis. However, a similar study at a much detailed spatial scale such as census tract or block group, hypothesis can be derived and practical results be achieved. Though in this present study the technique is used to investigate cancer mortality it can also be extended to include similar public health datasets. In future research we intend to study the effects of scale and discretization on the kind of association rules generated from mining public health, socioeconomic, and environmental datasets.

## Materials and methods

### Knowledge discovery and association rule mining

The concept of knowledge discovery is to extract implicit information in a dataset. Knowledge extracted from a dataset refers to a set of rules that are implicit, valid, novel, potentially useful, and those that are easily comprehensible by humans [29,45]. The process of extracting knowledge is an interactive and iterative procedure involving many tasks [29,45,46].

Association analysis is one of the most widely researched topics in data mining [30]. The main focus of association rule mining is to generate hypothesis rather than to test them as is commonly achieved using statistical techniques. Association rule mining, was first conceived and used for analyzing market-basket data to *mine *customer shopping patterns [31]. The idea was to find relations among the items (termed as predicates) purchased so that the customers could then be targeted for marketing specific products. An association rule typically consists of 3 parts – an antecedent (X), a consequent (Y), and a measure of the interestingness of the rule (*support*%, *confidence*%, *lift*), as represented in Equation 1.

X → Y (*support*%, *confidence*%, *lift*)     (1)

The antecedent and the consequent are a set of one or more predicates. The support of a rule measures the frequency of collective occurrence of all the antecedent and consequent predicates of a rule in the dataset. The confidence measures the frequency of occurrence of the consequent given the occurrence of the antecedent, while the lift measures the likelihood of the occurrence of the consequent given the antecedent. A lift value of 1 would indicate that the predicates expressed in the rule are independent of each other, while a value greater than 1 would indicate that an occurrence of the antecedent indicates a high probability for the occurrence of the consequent (positive correlation) whereas a lift value of less than 1 would indicate a negative correlation i.e., the occurrence of the antecedent discourages the occurrence of the consequent.

For example, consider a dataset composed of 100 records of cancer mortality and median income. Let 60 records indicate high rates of mortality, 50 records indicate low median income, and 40 records be comprised of both high rates of mortality and low median income values. Assuming the association analysis produces a rule linking low median income and high cancer mortality, the rule would be of the form shown in Equation 2:

High cancer mortality → Low median income (40%, 66.67%, 1.33)     (2)

An association rule as shown in Equation 2 is to be interpreted as areas that exhibit high rates of cancer mortality are characterized by low median income values. The support of a rule is calculated as the ratio of the number of records containing both antecedent (high cancer mortality) and consequent (low median income) to the total number of records in the dataset expressed as a percentage, i.e., (40/100)*100 = 40%. The confidence of a rule is the ratio of the number of records containing both antecedent and consequent to the number of records that contain the antecedent and is expressed as a percentage, i.e., (40/60)*100 = 66.67%. The lift is measured as the ratio of the probability of antecedent and consequent occurring together to the probability of antecedent and consequent occurring independently. In this case the lift is calculated as:



The lift value of 1.33 (greater than 1) indicates a positive correlation among low median income and high cancer mortality.

There have only been a few studies that applied association rule mining to extract spatial associations from socioeconomic data [15-17,47]. In the field of medicine and public health, association rule mining was used to uncover patterns from hospital infection control and public health surveillance data, resulting in discovery of patterns like increased resistance of the microbe *P. aeruginosa *to anti-microbials over a period of three months [18]. In a study on the factors related to heart disease, an association was found between the septo anterior region and left descending artery, implying that when there is a defect in the septo anterior region of the heart it is likely that the left descending artery is diseased [19,20]. It is noted that traditional multivariate statistical methods such as regression, canonical correlation analysis, and factor analysis could also be applied to analyze relationships [23-25,28]. Association rule mining, however, may be more efficient in generating hypotheses and revealing patterns that are not obvious (i.e., anomalies). With adequate visualization tools, the results from association rule mining can readily be shown and regions of high associations identified [48].

### Materials

The cancer mortality dataset used in this study was extracted from the Atlas of United States Mortality [10]. The dataset included gender and race specific mortality rates for the four most common types of cancer – colorectal cancer, lung cancer, breast cancer for women, and prostate cancer for men, aggregated over the years 1988 – 1992 at the Health Service Area (HSA) level and age-adjusted according to the 1940 U.S. standard million population. The mortality rates are reported per 100,000 of the population. The dataset only included those areas whose mortality rates were determined to be statistically reliable [10] in order to avoid spurious associations that might be a result of high cancer rates due to small numbers in death count. Because of the small number problem [33], which requires that there occur a minimum of 20 death cases for the resulting mortality rate to be considered statistically reliable, the HSA was chosen as the underlying unit of analysis as it provided for a compromise between having to choose a large area for a small time-period or a large time-period for a small area. In part, the decision to choose HSA level data was also due to the fact that it included a longitudinal time period centered on the 1990 census year and because of its availability to the authors. The mortality data was classified based on the International Classification of Diseases (ICD) 9 classification scheme. A HSA is a geographic area comprising of one or more contiguous counties delineated for the purposes of health planning. In all there were a total of 805 HSAs encompassing 3,141 counties in the U.S. based on the 1990 census information. Table 2 provides descriptive statistics for the reliable cancer mortality rates used in the current study.

The county-level socioeconomic data were obtained from the U.S. Census Bureau and the U.S. Bureau of Economic Analysis, and comprised five categories: family composition, education, housing, economic conditions, and occupation. A complete list of the variables used in this study is summarized in Table 1. These variables were chosen based in part on their ability to describe the living conditions of the general population and in part on having been used in area-based statistical studies involving the use of socioeconomic characteristics to describe public health [42,43]. Direct income measures were not used in the current study because the Census Bureau provides per capita and median income values for a county and these values cannot be aggregated to the HSA level. As such, measures of the availability of household plumbing facilities and households with no privately owned vehicles were used as surrogate measures of income and poverty levels. The county-level census and economic data were aggregated at the HSA level so that they can be analyzed together with the cancer mortality data. All socioeconomic variables used in the study were normalized based on their underlying universe value as reported in the 1990 US Census. The unemployment rate was expressed in percentage while the rest of socioeconomic variables were expressed as proportion. Table 3 provides for descriptive statistics for selected socioeconomic variables used in the current study.

### Methods

For association rule mining the Classification Based Association (CBA ver2.0) software was used [49,50]. A more up to date list of software for association analysis can be found at the KDNuggets website [51]. Compared to other association rule mining software CBA can be downloaded for free and provides a graphical user interface that is convenient to analyze the thousands of association rules extracted by the analysis. One disadvantage of the software is that it does not calculate the lift value and for the current study lift was calculated separately based on the values generated by the program. Magnum Opus is commercially available software from RuleQuest^® ^[52] and provides for such metrics as lift other than just the support and confidence values.

Since association rule mining requires categorical input data, each variable in the dataset was independently discretized into 5 quantile groups with each group containing about 20% of the records in the dataset. The cancer mortality rates that were classified by Pickle [10] as unreliable were excluded from the discretization process and marked as missing data. The five groups were labeled low, medium-low, medium, medium-high, and high respectively. A minimum support value of 10% generated a large number of association rules among the socioeconomic variables themselves, with only a small number of these rules including cancer mortality variables. Since the intent of the study was to find associations between the socioeconomic variables and cancer mortalities, the support value was iteratively decreased until a suitable number of rules consisting of such associations were generated. After several iterations, the minimum support level was set at 3% and a minimum confidence level of 40%. In other words, the probability that a particular rule occurs in the dataset was set to ≥ 0.03 and the minimum probability that the consequent predicates of a rule occurs given its antecedent predicates was set to ≥ 0.4.

The rationale for setting a minimum support value is only to limit the number of rules that are extracted. An association with a low support value would indicate an infrequent pattern that might not be interesting because of its very rare occurrence. Because in the present study the dataset was divided into five groups with each group consisting of about twenty percent of the data a high support value (close to and greater than 20) is not possible. The choice of minimum support and confidence values are subjective and should be based on the task relevancy. While a low support value might be useful in extracting patterns that might otherwise be disregarded, studies that are aimed at extracting patterns that are widely prevalent should consider a high support value.

## List of abbreviations

GIS – Geographic Information Systems

HSA – Health Service Area

CBA – Classification Based Association

ICD – International Classification of Diseases

## Competing interests

SV and NSNL have no competing interests in the current study.

## Authors' contributions

SV gathered the dataset and performed the analysis. NSNL provided critical review and input in the data analysis. Both the authors were involved in writing the manuscript.
